# Diagnostic yield of chromosomal microarray analysis and exome sequencing in fetuses with central nervous system anomalies, with long-term follow-up: a single-center study over a 17-year period

**DOI:** 10.1007/s00404-026-08481-5

**Published:** 2026-06-02

**Authors:** Karla Feodorovici, Hannah Klinkhammer, Brigitte Strizek, Ulrich Gembruch, Annegret Geipel

**Affiliations:** 1 Department of Obstetrics and Prenatal Medicine, University Hospital Bonn, Bonn, Germany; 2 Institute for Medical Biometry and Statistics, University Marburg, Marburg, Germany; 3 Institute for Genomic Statistics and Bioinformatics, University Hospital Bonn, Bonn, Germany; 4 Department of Obstetrics and Gynecology, University Hospital Bonn, Venusberg-Campus 1, 53127 Bonn, Germany

**Keywords:** Prenatal molecular genetics, Chromosomal microarray analysis, Exome sequencing, Fetal CNS anomalies, Soft marker, Minor and dynamic abnormalities, Neurodevelopmental outcome

## Abstract

**Objective:**

To assess the diagnostic yield of chromosomal microarray analysis (CMA) and exome sequencing (ES) in fetuses with central nervous system (CNS) anomalies across two study periods (January 2008 to June 2016 and July 2016 to December 2024), and to evaluate long-term outcomes.

**Methods:**

This retrospective study included cases with fetal CNS anomalies undergoing invasive testing by G-banded karyotyping and, in selected cases, additional CMA and/or ES. Fetuses were classified into four groups: isolated CNS malformations, isolated CNS plus (additional minor abnormalities and dynamic findings), complex CNS anomalies, and multisystem malformations.

**Results:**

Among 780 cases, causative findings were identified in 23.8% (25/105) by CMA and 39.0% (73/187) by ES. Use of CMA/ES increased in the second study period (16.3% vs. 59.7%), resulting in more pathogenic findings (26 vs. 72 cases), whereas diagnostic yield among tested cases decreased without significance. The highest ES yield was observed in multisystem CNS anomalies; isolated CNS plus cases showed higher yields than truly isolated CNS cases. In an exploratory subgroup analysis, isolated CNS plus cases with polyhydramnios had a higher CMA/ES yield than truly isolated cases. Among 91 live-born children with follow-up, 49.5% (45/91) had global developmental delay, which was more frequent with pathogenic findings (90.0%, 18/20; *p* = 0.002) and increasing anomaly complexity (11.1 to 81.8%; *p* < 0.001).

**Conclusion:**

Uptake of CMA and ES increased over time and resulted in more etiologic diagnoses. In tested cases, both methods provided meaningful diagnostic yield in fetuses with CNS anomalies and may contribute to prognostic assessment and prenatal counseling.

**Supplementary Information:**

The online version contains supplementary material available at 10.1007/s00404-026-08481-5.

## What does this study add to the clinical work?

**Table:** 

We implemented an expanded classification model to distinguish between truly and non-truly isolated fetal central nervous system (CNS) anomalies and to facilitate phenotypic stratification for genetic testing.	
Our data show that the increased uptake of chromosomal microarray analysis and exome sequencing over time led to more etiological diagnoses, while the diagnostic yield remained dependent on the phenotype.	
The findings of the present study support consideration of molecular genetic testing not only in multisystem anomalies, but also in selected CNS-confined anomalies.	
Long-term follow-up data suggest that both anomaly complexity and pathogenic genetic findings are relevant for prognostic assessment and prenatal counseling.	

## Introduction

Central nervous system (CNS) malformations belong to the most prevalent congenital anomalies, with an incidence of around 1.5 per 1000 live births and 3–6% of stillbirths [1]. Studies with long-term follow-up suggest an even higher prevalence of up to 1 per 100 live births [2]. Prenatal assessment of CNS anomalies is most reliable after 20–22 weeks of gestation, whereas malformations of cortical development may become apparent later.[3, 4] CNS malformations have a broad etiological spectrum, with genetic causes playing a major role.[5, 6] Prenatal genetic diagnostics have expanded considerably in recent years. G-banded karyotyping, used as a first-tier test, detects chromosomal abnormalities in 11.3% of fetuses with CNS anomalies.[7] More recent approaches, including chromosomal microarray analysis (CMA) and exome sequencing (ES), enable the detection of submicroscopic copy number variants and monogenic disorders.[8–13] CMA has been reported to provide a diagnostic yield of 6.7%, while ES yields range from 7.2 to 19.0%, depending on phenotypic complexity and the presence of extra-CNS anomalies.[14–16] Several studies have described associations between dynamic findings, such as amniotic fluid abnormalities and fetal growth restriction (FGR), as well as minor anomalies or soft markers.[17–22] However, it remains unclear whether these findings increase the likelihood of an abnormal genetic diagnosis.

The outcome of fetuses with CNS anomalies is highly variable, ranging from normal neurodevelopment to severe intellectual and motor impairment or even death.[23–25] Similar prenatal phenotypes may arise from different etiologies, whereas distinct disorders may present with overlapping features. Identification of an underlying genetic cause may improve prognostic assessment and prenatal counseling; in selected CNS anomalies, pathogenic genetic findings have been associated with poorer neurodevelopmental outcome.[26, 27].

The aim of this study was to assess the diagnostic yield of CMA and ES in tested cases across different subgroups of fetal CNS malformations in two study periods (January 2008 to June 2016 and July 2016 to December 2024). In addition, we explored the association of minor and dynamic anomalies in otherwise isolated cases and assessed long-term outcomes.

## Methods

This retrospective study was conducted at a tertiary referral center for Obstetrics and Prenatal Medicine in Bonn, Germany. Pregnancies with fetal structural CNS anomalies and invasive genetic testing between January 2008 and December 2024 were included. Isolated neural tube defects, isolated choroid plexus cysts, infections, hemorrhages, vascular events, twin reversed arterial perfusion sequence, and brain anomalies secondary to fetofetal transfusion syndrome were excluded [5, 17, 28]. All patients underwent detailed high-resolution fetal ultrasound by an experienced fetal medicine specialist. Findings were recorded in a dedicated database (ViewPoint). Fetal magnetic resonance imaging was performed in selected cases. Soft markers (echogenic intracardiac focus, echogenic bowel, nuchal edema, lateral neck cysts, absent/hypoplastic nasal bone, short femur, pyelectasis, tricuspid insufficiency) and minor lesions (single umbilical artery, persistent right umbilical vein, varix of the umbilical vein, choroid plexus cyst, dilated gallbladder, small muscular ventricular septal defect, aberrant right subclavian artery, persistent left superior vena cava, right aortic arch, mild cardiomegaly) were recorded separately.

Cases were classified according to imaging findings into one of four main groups: “isolated CNS” (isolated CNS anomaly), “isolated CNS plus” (isolated CNS anomaly with at least one of the following: FGR (including estimated fetal weight (EFW) between the 3rd and 10th percentile or ≤ 3rd percentile with underlying uteroplacental dysfunction), amniotic fluid alterations, minor lesions or soft markers), “complex CNS” (multiple CNS anomalies), or “multisystem CNS” (associated with other organ malformations). Cases of polyhydramnios secondary to gestational diabetes were not included in the isolated CNS plus group. In fetuses with an isolated CNS anomaly, severe FGR (EFW ≤ 3rd percentile) in the absence of Doppler signs of uteroplacental dysfunction was regarded as an additional anomaly, hence these fetuses were classified as “multisystem CNS”. Early- and late-onset FGR were defined as FGR occurring before or at/after 32 weeks of gestation, respectively, according to an international Delphi consensus [29]. Structural CNS anomalies were grouped as ventriculomegaly, posterior fossa malformations, brainstem anomalies, midline defects, cortical disorders, intracranial cysts (unrelated to the posterior fossa), and tumors. Ventriculomegaly was classified as mild (10–12 mm), moderate (> 12–15 mm), or severe (> 15 mm), based on previously published thresholds and established clinical recommendations [30, 31].

**Fig. 1:**
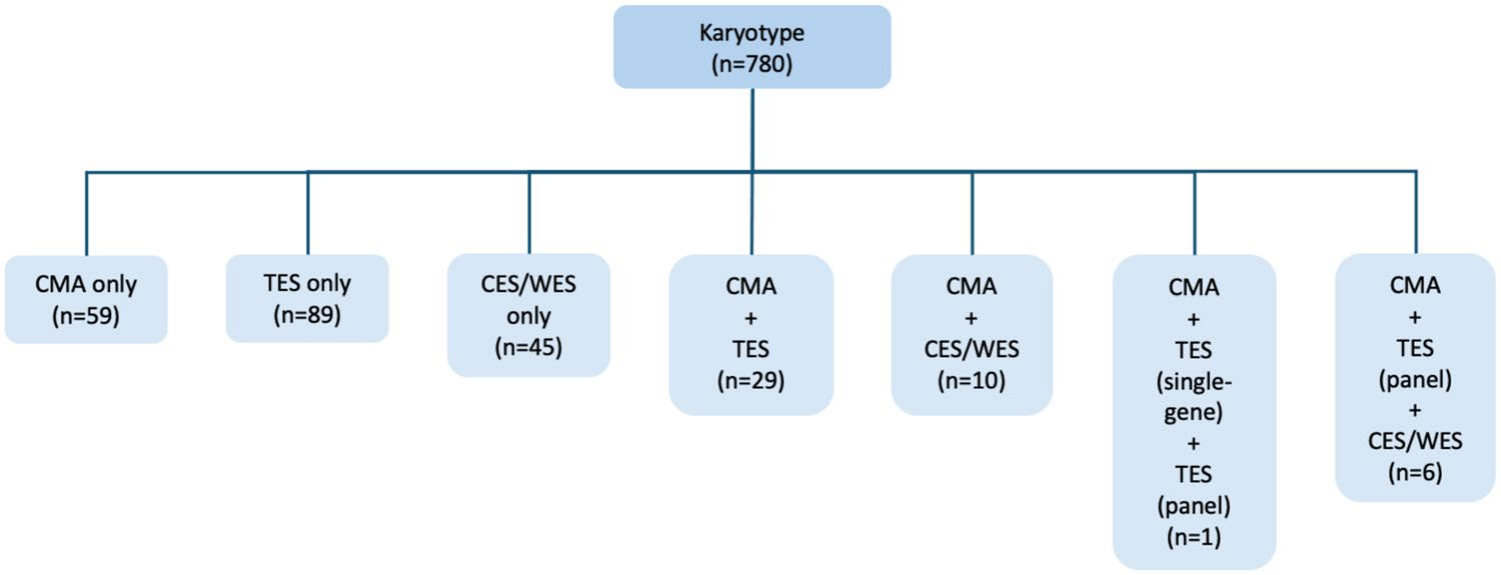
Overview of genetic diagnostic approaches applied in the study cohort (n = 780). All cases initially underwent karyotype analysis, with additional genetic investigations performed in selected cases, including CMA and different exome sequencing approaches. Only a minority of investigations were performed postnatally, including karyotype (7/780), CMA (13/105), and ES (16/187); these are not displayed separately in the diagram. *CMA*, chromosomal microarray analysis, *CES*, clinical exome sequencing, *ES* exome sequencing, *TES* targeted exome sequencing, *WES* whole-exome sequencing

Genetic testing comprised G-banded karyotyping in the entire cohort and, in selected cases, CMA and ES, the latter encompassing targeted ES (single-gene and gene panel analyses), clinical exome sequencing, and whole-exome sequencing (Fig. 1). CMA and ES were performed selectively based on clinical indication and availability. Findings were considered diagnostic if classified as pathogenic or likely pathogenic (P/LP) according to American College of Medical Genetics and Genomics criteria and consistent with the fetal phenotype.

Both prenatal and postnatal genetic diagnoses were included in the calculation of diagnostic yield. For combined CMA/ES analyses, denominators were based on the total number of CMA and ES tests performed within each subgroup rather than the number of individual cases. As CMA and ES were partly performed sequentially rather than simultaneously, some cases contributed to both groups; therefore, overlap in the denominator cannot be excluded.

To compare diagnostic findings over time, the cohort was divided into two time periods of equal duration: period A (January 2008 to June 2016) and period B (July 2016 to December 2024).

Outcomes were classified as termination of pregnancy (TOP), intrauterine fetal demise, liveborn with neonatal death, infant death and death after the first year of life. Outcome data were obtained from delivery, neonatal, and pediatric records and autopsy findings. Long-term follow-up was assessed by parental contact and review of pediatric and neuropediatric reports using a standardized questionnaire addressing developmental delay, motor and cognitive impairment, speech, hearing and visual disorders, need for medical aids and supportive measures.

Statistical analyses were performed using R 4.3.2. Continuous variables were compared by ANOVA and categorical variables by χ^2^ or Fisher’s exact test, as appropriate. *p* < 0.05 was considered statistically significant. Diagnostic yield was defined as the proportion of P/LP results among completed tests.

## Results

780 fetuses met the inclusion criteria, including 5.9% multiple pregnancies. Mean maternal age at diagnosis was 31.9 ± 5.7 (16–51) years, with 33.8% aged ≥ 35 years. Mean gestational age at diagnosis was 21.6 ± 4.7 (11 + 3 to 36 + 0) weeks. Baseline characteristics and case distribution across the four study groups are shown in Table 1.

**Table 1:** Baseline characteristics

		Total	Isolated CNS	Isolated CNS plus	Complex CNS	Multisystem CNS	*p*-value
Number of fetuses		780	126 (16.2%)	52 (6.7%)	124 (15.9%)	478 (61.3%)	
Consanguinity		31 (4%)	3 (2.4%)	1 (1.9%)	2 (1.6%)	25 (5.2%)	0.210^c^
Positive genetic family history		32 (4.1%)	4 (3.2%)	1 (1.9%)	9 (7.3%)	18 (3.8%)	0.309^c^
Amniotic fluid	Oligohydramnios	53 (6.8%)	–	4 (7.7%)	6 (4.8%)	43 (9%)	0.335^c^
	Polyhydramnios	118 (15.1%)	–	17 (32.7%)	8 (6.5%)	93 (19.5%)	< 0.001^b,g−i^
FGR (early-onset)		210 (26.9%)	–	11 (21.2%)	12 (9.7%)	187 (39.1%)	< 0.001^b,h,i^
Minor lesions		201 (25.8%)	–	20 (38.5%)	13 (10.5%)	168 (35.1%)	< 0.001^b,g,i^
Soft markers		123 (15.8%)	–	15 (28.8%)	5 (4%)	103 (21.5%)	< 0.001^b,g,i^
Prenatal MRI	Performed	143 (18.3%)	30 (23.8%)	14 (26.9%)	45 (36.3%)	54 (11.3%)	< 0.001^b,e,f,h,i^
	Diagnostic yield	37 (25.9%)	4 (13.3%)	2 (14.3%)	14 (31.1%)	17 (31.5%)	0.177^c^
*Genetic testing and pathologic findings*^a^							
Karyotype		189/780 (24.2%)	4/126 (3.2%)	15/52 (28.8%)	7/124 (5.6%)	163/478 (34.1%)	< 0.001^c,d,f,g,i^
CMA		25/105 (23.8%)	3/21 (14.3%)	3/7 (42.9%)	2/16 (12.5%)	17/61 (27.9%)	0.256^c^
ES		73/187 (39%)	3/33 (9.1%)	3/6 (50%)	8/34 (23.5%)	59/114 (51.8%)	< 0.001^c,d,f,i^
*Outcome*							
TOP		505 (64.7%)	62 (49.2%)	28 (53.8%)	83 (66.9%)	332 (69.5%)	< 0.001^b,e,f,h^
IUFD		21 (2.7%)	2 (1.6%)	1 (1.9%)	2 (1.6%)	16 (3.3%)	0.697^c^
Live births		221 (28.3%)	54 (42.9%)	19 (36.5%)	34 (27.4%)	114 (23.8%)	< 0.001^c,e,f,h^
Unknown outcome		33 (4.2%)	8 (6.3%)	4 (7.7%)	5 (4%)	16 (3.3%)	
NND		26/221 (11.8%)	–	–	–	26/114 (22.8%)	
Infant death		10/221 (4.5%)	–	–	–	10/114 (8.8%)	
Death ≥ 1 yr		5/221 (2.3%)	–	1/19 (5.3%)	1/34 (2.9%)	3/114 (2.6%)	

Neonatal death was defined as death within 28 days after birth and infant death as death between day 29 and 1 year

*CMA* chromosomal microarray analysis, *CNS* central nervous system, *ES* exome sequencing, *FGR* fetal growth restriction, *IUFD* intrauterine fetal demise, *MRI* magnetic resonance imaging, *NND* neonatal death, *TOP* termination of pregnancy, *yr* year

^a^Pathogenic or likely pathogenic results devided by total number of genetic investigations performed

*p*-value calculated by Chi-square test^b^, Fisher's test^c^. *p*-value < 0.05 in pairwise testing: Isolated vs. Isolated plus^d^, Isolated vs. Complex^e^, Isolated vs. Multisystem^f^, Isolated plus vs. Complex^g^, Isolated plus vs. Multisystem^h^, Complex vs. Multisystem^i^

**Fig. 2:**
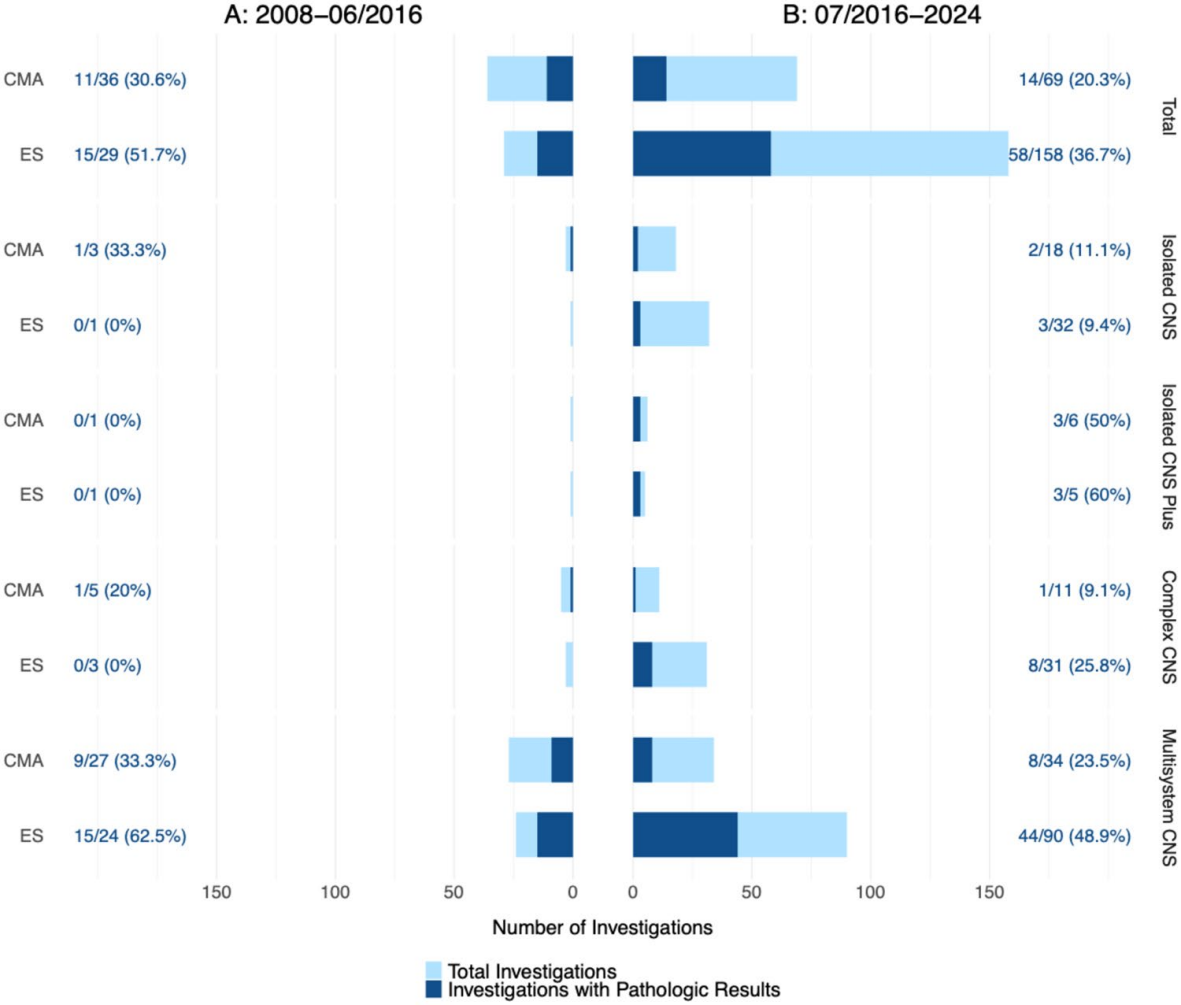
Molecular genetic diagnostic yield according to period (A and B). Each row shows the number and percentage of pathological results among the tested cases. *CNS* central nervous system, *CMA* chromosomal microarray analysis, *ES* exome sequencing

G-banded karyotyping was performed by chorionic villous sampling in 7.6% (59/780), amniocentesis in 87.8% (685/780), percutaneous umbilical blood sampling in 3.7% (29/780), and postnatal testing in 0.9% (7/780) of cases. Figure 1 illustrates the different molecular genetic testing pathways in our cohort. Genetic investigations were compared between period A (n = 400) and period B (n = 380); subgroup distribution was comparable across both periods (n = 60 vs. 66 for isolated CNS, 32 vs. 20 for isolated CNS plus, 61 vs. 63 for complex CNS, and 247 vs. 231 for multisystem CNS, respectively). G-banded karyotyping detected abnormalities in 21.5% (86/400) in period A and 27.1% (103/380) in period B (*p* = 0.08). The proportion of cases undergoing CMA and/or ES increased from 16.3% in period A to 59.7% in period B (*p* < 0.001) (Fig. 2). CMA nearly doubled (n = 36 vs. n = 69), whereas ES increased more than fivefold (n = 29 vs. n = 158). In period A, CMA/ES was predominantly performed in multisystem cases, whereas in the second period its use extended across all subgroups. This resulted in a lower diagnostic yield among tested cases (CMA: 30.6% vs. 20.3%, *p* = 0.35; ES: 51.7% vs. 36.7%, *p* = 0.19), while the absolute number of genetic diagnoses rose (26 cases in period A vs. 72 cases in period B), mainly driven by ES use. Mean gestational age at the time an abnormal genetic result became available was 21.6 (12–37) weeks for karyotyping and 25.4 (15–33) weeks for CMA/ES. Across the entire study period, 23.8% of CMA-tested cases and 39.0% of ES-tested cases had a P/LP finding (Table 1).

The highest ES yield was observed in the multisystem group and the lowest in the isolated CNS group (51.8% vs. 9.1%, respectively; *p* < 0.001). The isolated CNS plus group showed higher proportions of diagnostic findings than the isolated CNS group (karyotyping: 28.8% vs. 3.2%, *p* < 0.001; when CMA and ES were analyzed together based on the total number of tests performed: 46.2%, 6/13 vs. 11.1%, 6/54; *p* = 0.01). Isolated CNS plus cases with polyhydramnios presented a higher diagnostic yield of CMA and/or ES than truly isolated cases (80.0%, 4/5 vs. 11.1%, 6/54; *p* = 0.002). All four tested cases with polyhydramnios had a pathogenic finding, including one identified by CMA and three by ES. Given the small number of cases, however, this finding should be interpreted as exploratory. Supplementary Table S1 provides a detailed overview of CNS-confined cases with abnormal molecular genetic findings.

Supplementary Table S2 presents the specific fetal CNS anomalies in our cohort, along with their distribution across study groups.

Table 2 summarizes pathogenic findings by CNS anomaly type; several subgroup analyses were based on small numbers and should therefore be regarded as exploratory.

**Table 2:** Incidence of pathogenic/likely pathogenic results according to different types of CNS anomalies

CNS anomaly	Genetics	Total	Isolated CNS	Isolated CNS plus	Complex CNS	Multisystem CNS	*p*-value^a^
Ventriculomegaly (n = 481)	Karyotype	79/481 (16.4%)	1/98 (1%)	12/40 (30%)	5/92 (5.4%)	61/251 (24.3%)	< 0.001^d,f,g,i^
	CMA	11/60 (18.3%)	1/14 (7.1%)	2/5 (40%)	0/10 (0%)	8/31 (25.8%)	0.103
	ES	49/134 (36.6%)	3/29 (10.3%)	2/5 (40%)	3/26 (11.5%)	41/74 (55.4%)	< 0.001^f,i^
Midline defects (n = 365)	Karyotype	83/365 (22.7%)	1/47 (2.1%)	0/15 (0%)	5/79 (6.3%)	77/224 (34.4%)	< 0.001^f,h,i^
	CMA	13/58 (22.4%)	2/10 (20%)	3/6 (50%)	1/12 (8.3%)	7/30 (23.3%)	0.281
	ES	35/92 (38%)	0/11 (0%)	0/3 (0%)	5/22 (22.7%)	30/56 (53.6%)	< 0.001^f,i^
Posterior fossa anomalies (n = 270)	Karyotype	61/270 (22.6%)	2/16 (12.5%)	2/6 (33.3%)	4/62 (6.5%)	53/186 (28.5%)	< 0.001^i^
	CMA	8/42 (19%)	0/4 (0%)	–	0/6 (0%)	8/32 (25%)	0.313
	ES	35/75 (46.7%)	0/3 (0%)	1/1 (100%)	6/16 (37.5%)	28/55 (50.9%)	0.198
Cortical malformations (n = 190)	Karyotype	24/190 (12.6%)	0/1 (0%)	1/1 (100%)	2/73 (2.7%)	21/115 (18.3%)	< 0.001^ g,i^
	CMA	9/43 (20.9%)	0/1 (0%)	–	3/14 (21.4%)	6/28 (21.4%)	1.000
	ES	31/71 (43.7%)	0/1 (0%)	–	9/29 (31%)	22/41 (53.7%)	0.090
Others^b^ (n = 51)	Karyotype	7/51 (13.7%)	–	0/1 (0%)	2/19 (10.5%)	5/31 (16.1%)	0.737
	CMA	0/9 (0%)	–	–	0/1 (0%)	0/8 (0%)	
	ES	7/14 (50%)	–	–	0/4 (0%)	7/10 (70%)	
Intracranial cysts^c^ (n = 36)	Karyotype	1/36 (2.8%)	0/1 (0%)	–	1/20 (5%)	0/15 (0%)	1.000
	CMA	1/6 (16.7%)	–	–	1/5 (20%)	0/1 (0%)	
	ES	3/12 (25%)	–	–	0/5 (0%)	3/7 (42.9%)	
Brain stem anomalies (n = 9)	Karyotype	1/9 (11.1%)	–	–	0/3 (0%)	1/6 (16.7%)	
	CMA	0/3 (0%)	–	–	0/1 (0%)	0/2 (0%)	
	ES	3/5 (60%)	–	–	–	3/5 (60%)	

The total number of anomalies exceeds the number of fetuses (n = 780) as fetuses could present with more than one anomaly

*CMA* chromosomal microarray analysis, *CNS* central nervous system, *ES* exome sequencing

^a^*p*-values were calculated by Fisher's exact test

^b^including encephaloceles, hydranencephaly and brain tumors

^c^excluding cysts of the posterior fossa

*p*-value < 0.05 in pairwise testing: Isolated vs. Isolated plus^d^, Isolated vs. Complex^e^, Isolated vs. Multisystem^f^, Isolated plus vs. Complex^g^, Isolated plus vs. Multisystem^h^, Complex vs. Multisystem^i^

In total, 81 P/LP variants involving 54 genes were detected. The most frequently affected genes and associated conditions were *L1CAM* (11.1%, 9/81; L1 syndrome/X-linked hydrocephalus), *POMT1* and *PAFAH1B1* (each 4.9%, 4/81; Walker-Warburg syndrome and Miller-Dieker syndrome, respectively), and *FGFR2*, *TUBA1A*, *CEP290*, *PDHA1*, and *PTPN11* (each 3.7%, 3/81; Apert syndrome, lissencephaly type 3, Meckel-Gruber syndrome, pyruvate dehydrogenase deficiency, and Noonan syndrome, respectively).

In the isolated CNS and isolated CNS plus groups, the associated genetic disorders were NF1 microduplication syndrome (*NF1*; 22.2%, 2/9), followed by L1 syndrome *(L1CAM)*, Kabuki syndrome *(KMT2D)*, congenital hydrocephalus-5 *(SMARCC1)*, lissencephaly type 3 *(TUBA1A)*, Bohring-Opitz syndrome *(ASXL1)*, spastic paraplegia type 47 *(AP4B1)*, and Imagawa-Matsumoto syndrome *(SUZ12)* (each 11.1%, 1/9). In the complex and multisystem groups, the most common genetic syndromes were L1 syndrome (*L1CAM*; 11.1%, 8/72), Miller-Dieker syndrome *(PAFAH1B1)* and Walker-Warburg syndrome *(POMT1)* (each 5.6%, 4/72).

Overall, 64.7% of pregnancies ended in TOP, which was significantly associated with anomaly complexity (Table 1). Termination was requested more frequently in cases with pathogenic CMA/ES findings than in those with inconspicuous results (65.6%, 63/96 vs. 35.0%, 64/183; *p* < 0.001).

Long-term outcome was available in 41.2% of live births (n = 91), ranging from one month to 16 years (mean 5.2 years). Within the follow-up cohort, subgroup distribution was 29.7% (27/91) isolated CNS, 11.0% (10/91) isolated CNS plus, 23.1% (21/91) complex CNS, and 36.3% (33/91) multisystem CNS.

A normal outcome without impairments or need for supportive aids was reported in 22.0% (20/91), most frequently in isolated CNS (40.7%, 11/27) and isolated CNS plus cases (50.0%, 5/10), but less often in complex CNS (14.3%, 3/21) and multisystem CNS cases (3.0%, 1/33).

An abnormal outcome was observed in 78.0% (71/91) of children; notably, 49.5% (45/91) had global developmental delay (GDD), including motor and cognitive impairment. The frequency of GDD increased with anomaly complexity, from 11.1% (3/27) in isolated CNS to 40.0% (4/10) in isolated CNS plus, 52.3% (11/21) in complex CNS, and 81.8% (27/33) in multisystem CNS cases (*p* < 0.001). Speech disorders were observed in 44.0% (40/91), visual impairment in 29.7% (27/91), and hearing deficits in 7.7% (7/91). Epilepsy was present in 23 children. Supportive measures, including inclusive daycare or schooling, occupational therapy, physiotherapy, or speech therapy, were required in 70.3% (64/91), and 51.6% (47/91) relied on medical aids.

In cases undergoing CMA and/or ES, the distribution of CNS anomaly subgroups did not differ significantly between those with and without available follow-up (*p* = 0.29), although complex CNS anomalies were numerically more frequent in the follow-up group (Table 3). Among the 56 children with follow-up who underwent CMA and/or ES, 35.7% (20/56) had a P/LP finding. GDD was observed in 90.0% (18/20) of children with a P/LP finding, compared with 47.2% (17/36) of those with negative molecular findings (*p* = 0.002).

**Table 3:** Comparison of cases with performed CMA and/or ES with available long-term follow-up (n = 56) and cases with performed CMA and/or ES without follow-up data (n = 44)

	Isolated CNS		Isolated CNS plus		Complex CNS		Multisystem CNS	
Follow-up available	Yes (n = 12)	No (n = 10)	Yes (n = 3)	No (n = 2)	Yes (n = 13)	No (n = 4)	Yes (n = 28)	No (n = 28)
**Normal CMA/ES**	11	10	2	1	12	1	11	21
Normal outcome	5	–	1	–	3	–	0	–
Abnormal outcome	6	–	1	–	9	–	11	–
including GDD	1	–	1	–	6	–	9	–
**Abnormal CMA/ES**	1	0	1	1	1	3	17	7
Normal outcome	0	–	0	–	0	–	0	–
Abnormal outcome	1	–	1	–	1	–	17	–
including GDD	0	–	1	–	1	–	16	–

Data are presented as n

*CMA* chromosomal microarray analysis, *CNS* central nervous system, *ES* exome sequencing, *GDD* global developmental delay

Within complex and multisystem CNS cases with GDD who underwent CMA and/or ES, P/LP variants were more frequently identified in multisystem than in complex CNS cases (64.0%, 16/25 vs. 14.3%, 1/7; *p* = 0.02), although the small number of cases warrants cautious interpretation.

## Discussion

The uptake of molecular genetic testing, particularly ES, increased markedly over time in fetuses with CNS anomalies, resulting in more etiologic diagnoses, while the diagnostic yield among tested cases remained stable, with a slight but non-significant decline. This may reflect broader use of CMA and ES in less selected phenotypes, whereas earlier testing was mainly reserved for severe multisystem anomalies with a higher pretest probability. Accordingly, the reported yields should be interpreted as yields among tested cases rather than cohort-wide detection rates. These findings align with meta-analytic data indicating no clear increase in yield by publication year despite rising test uptake, and are further supported by evolving recommendations, changes in clinical practice, and advances in phenotype–genotype correlations.[32–37] Stratified analyses did not indicate a systematic overestimation of the overall diagnostic yield due to inclusion of postnatal cases. However, this interpretation is limited by the small number of postnatal cases. The comparatively limited use of prenatal CMA in our cohort may partly reflect reimbursement policies in Germany, where CMA is often not reimbursed, in contrast to G-banded karyotyping and ES. Furthermore, the clinical impact of genetic testing depends not only on the diagnostic yield, but also on the timing of result availability during pregnancy. In our cohort, abnormal karyotype results became available at a mean gestational age of 21.6 weeks, compared with 25.4 weeks for CMA/ES. This difference may be relevant in the context of time-sensitive prenatal counseling and decision-making.

A pathogenic genetic result was associated with a higher rate of TOP, suggesting that molecular findings contributed to parental decision-making [38, 39]. As ES is increasingly performed earlier in gestation and with shorter turnaround times, its clinical impact may become even greater.[40, 41] Blayney et al. reported high TOP rates in fetuses with CNS anomalies even after negative ES, exceeding those reported in recent single-center cohorts, including our own.[38, 39] These differences may reflect variation in phenotype severity, cultural factors, and legal regulations.

The majority of studies examining the genetic basis of fetal malformations classify cases as isolated, complex, or multisystem.[7, 14, 39] As expected, the likelihood of a pathogenic genetic finding increases with anomaly number and complexity.[8, 14, 39, 42] Notably, diagnostically meaningful yields were also observed in selected CNS-confined phenotypes. In particular, the isolated CNS plus group showed higher diagnostic yields than the truly isolated CNS group, suggesting that additional minor abnormalities, soft markers, FGR, or amniotic fluid abnormalities may increase the likelihood of an underlying genetic etiology. This is consistent with previous studies linking dynamic findings and minor anomalies to higher rates of genetic abnormalities.[17–22] The high molecular diagnostic yield observed in isolated CNS plus cases with unexplained polyhydramnios is noteworthy, but should be interpreted as exploratory due to the small number of cases. Rather than indicating a definitive subgroup effect, this finding suggests that apparently isolated CNS anomalies accompanied by additional dynamic findings may warrant closer consideration for molecular testing.

The overall diagnostic yields observed in the present cohort were relatively high compared with many previously published series for both CMA and ES [14–16, 43–46]. This may partly reflect the tertiary referral setting, selective cohort composition, exclusion of low-yield entities, as well as differences in testing indications and case selection across studies. Taken together, these findings support targeted genetic testing strategies, in line with recent systematic review and meta-analysis evidence.[35].

Prognostic evaluation is particularly challenging in CNS-confined anomalies, whereas multisystem abnormalities are generally associated with poorer outcomes. Molecular genetic testing may therefore add clinically relevant information for prognostic stratification. In support of this, a previous study reported poorer neurodevelopmental outcomes in children with isolated ventriculomegaly and prenatally identified pathogenic variants than in those without an underlying genetic diagnosis.[47] In our cohort, the CNS-confined groups (isolated CNS, isolated CNS plus, and complex CNS) showed meaningful diagnostic yields with CMA and ES, indicating that the potential value of genetic testing is not limited to multisystem phenotypes.

Children with congenital CNS malformations are at increased risk of adverse neurodevelopmental outcomes, yet data linking prenatal CNS findings with long-term postnatal development remain limited. Similar to previous reports, only a minority of live-born children in our cohort showed a normal outcome.[23] Prenatal counseling can be challenging, as prognosis depends on the type and extent of the malformation as well as the underlying etiology, while many syndromic disorders exhibit marked phenotypic variability. Data on the relationship between genetic findings and long-term neurodevelopmental outcome in fetuses with CNS anomalies remain scarce. In our cohort, the frequency of GDD increased with anomaly complexity and was higher in children with a P/LP finding than in those with negative molecular findings, suggesting that genetic findings may contribute to prognostic stratification. However, a normal molecular genetic result should not be interpreted as reassuring per se, as adverse outcomes may also occur in children with complex CNS malformations of non-genetic origin.

These findings should be interpreted in the context of the study design. Follow-up was available for only a subset of live-born children, introducing the possibility of selection bias, although no significant difference in subgroup distribution was observed between children with and without follow-up among cases undergoing CMA and/or ES.

Taken together, our findings suggest that both phenotypic complexity and genetic findings are relevant to long-term outcome, although prognostic conclusions should be drawn cautiously. These data nevertheless provide clinically relevant information for prenatal counseling and underscore the need for further studies with more systematic follow-up.

This study has several limitations, including its retrospective single-center design and the non-uniform use of genetic testing over time, partly related to reimbursement issues. Several subgroup analyses were based on small numbers and no correction for multiple comparisons was applied; these findings should therefore be interpreted cautiously and considered exploratory. Furthermore, the association between P/LP findings and GDD was not adjusted for phenotypic complexity, which may represent a relevant confounder. Due to the limited follow-up sample size, multivariable analyses were not feasible. This finding should therefore be interpreted with caution and warrants confirmation in future studies with larger cohorts. Strengths of this study include the comparatively large cohort of fetal CNS anomalies, the expanded classification model incorporating minor abnormalities, amniotic fluid alterations, and FGR, and the availability of long-term follow-up data.

In conclusion, the use of molecular genetic testing increased markedly from 16.3% to 59.7% between 2008–06/2016 and 07/2016–2024, resulting in a substantial rise in etiologic diagnoses. CMA and ES provided diagnostic value not only in multisystem anomalies, but also in selected CNS-confined phenotypes. Finally, anomaly complexity and pathogenic genetic findings were both associated with poorer long-term outcomes, underscoring their relevance for prenatal counseling and prognostic assessment.

## Supplementary Information

Below is the link to the electronic supplementary material.

Supplementary file1 (DOCX 307 KB)

## Data Availability

The data that support the findings of this study are available from the corresponding author upon reasonable request.
