# Supplementary material for: Diagnostic yield of chromosomal microarray analysis and exome sequencing in fetuses with central nervous system anomalies, with long-term follow-up: a single-center study over a 17-year period
Source: Arch Gynecol Obstet. 2026 Jun 2;313(1):198. doi: 10.1007/s00404-026-08481-5 (PMC13230342; doi:10.1007/s00404-026-08481-5)
Supplement: Supplementary file 1 — Supplementary file1 (DOCX 307 KB) [file 404_2026_8481_MOESM1_ESM.docx]

| **Table S1** - Case series presentation: Isolated CNS, isolated CNS plus and complex CNS cases with pathogenic/likely pathogenic molecular genetic findings. | | | | | | | | | |
| --- | --- | --- | --- | --- | --- | --- | --- | --- | --- |
| Case | Classification | Consang. | Sex | GA at Dx (weeks) | Phenotype | Genetic analysis | GA at genetic Dx (weeks) | Syndrome/disorder | Outcome |
| 1 | Isolated CNS | No | F | 21+3 | pACC | CMA: inversionduplication/-deletion 8p, 46,XX, dup(8)(p11.2->p23::p23->qter), ORPHA #96092 | 24+4 | 8p inverted duplication/deletion syndrome | TOP |
| 2 | Isolated CNS | No | M | 19+6 | Hydrocephalus internus, dichorionic-diamniotic twin | L1CAM-sequencing: inconspicuous. CMA: het (mat) 17q13.3 duplication (3 copies, 551 kb), OMIM #513215 | 26+5 | 17q13.3 microduplication syndrome | TOP |
| 3 | Isolated CNS | No | F | 22+1 | ACC | CMA: het 17q11.2 duplication(1.3 Mb), including NF1-gene, OMIM #618874 | 25+2 | NF1 microduplication syndrome | TOP |
| 4 | Isolated CNS | No | M | 20+6 | Hydrocephalus internus | Panel (270 genes): hemi L1CAM mutation (c.694+1G>A), OMIM #307000 | - | L1 syndrome | TOP |
| 5 | Isolated CNS | No | F | 17+5 | Hydrocephalus internus, dichorionic-diamniotic twin | Trio ES: het (mat) SMARCC1 mutation (c.585delT; p.Phe196Leufs*26 (het), NM_003074.4) | - | Congenital hydrocephalus-5 | Selective fetocide 31+5 |
| 6 | Isolated CNS | No | F | 25+5 | Hydrocephalus internus | Trio ES: het(mat) KMT2D mutation (c.16498C>T; p.Arg5500Trp), OMIM #147920 | 29+6 | Kabuki syndrome | Live birth 38+3, shunting 5 days postnatally. Follow up at 2 years: mild cognitive delay. |
| 7 | Isolated CNS plus | No | M | 22+0 | ACC with mild VM, pyelectasis | CMA: het 5p15.33p14.1 terminal deletion, OMIM #123450 | - | Cri-du-chat syndome | TOP |
| 8 | Isolated CNS plus | No | M | 26+1 | Macrosomic fet with ACC, polyhydramnios | CMA: 17q11.2 deletion, including NF1- and SUZ 12-genes, OMIM #613675 | - | NF1 microdeletion- and Imagawa-Matsumoto syndrome | TOP |
| 9 | Isolated CNS plus | No | F | 21+5 | Severe cerebellar hypoplasia, polyhydramnios | Trio ES: het (dn) TUBA1A mutation (C.521C>T p. (Ala174Val)), OMIM #611603 | 24+3 | TUBA1A-associated lissencephaly (lissencephaly 3) | TOP |
| 10 | Isolated CNS plus | No | M | 21+3 | Moderate VM (postnatally resolved), polyhydramnios | CMA: inconspicuous. Postnatal ES: ASXL1 mutation (20q11.21), OMIM #605039 | Postnatal | Bohring-Opitz syndrome | Live birth 37+4, LTFU |
| 11 | Isolated CNS plus | Yes | M | 18+2 | Hydrocephalus internus, polyhydramnios | ES: hom AP4B1 mutation, OMIM #614066 | - | Spastic paraplegia type 47 | TOP |
| 12 | Isolated CNS plus | No | M | 22+3 | Moderate VM, ACC, FGR | CMA: 14q11.2q21.1 deletion, OMIM #613457 | Postnatal | 14q11.2 microdeletion syndrome | Live birth 26+5, child died at the age of one year. GDD, epilepsy. |
| 13 | Complex CNS | No | M | 27+1 | Microcephaly, lissencephaly, supratentorial arachnoid cyst | CMA: deletion X-chromosome (q21.1), exons 35-41 | 29+1 | Becker-Kiener muscular dystrophy | TOP |
| 14 | Complex CNS | No | F | 29+2 | Pachygyria, CC dysgenesis, FGR, polyhydramnios | CMA: het(mat) deletion 17p13.3, LIS1 (PAFAH1B1), OMIM #247200 | - | Miller-Dieker syndrome | TOP |
| 15 | Complex CNS | No | M | 23+6 | Blake's pouch cyst, right cerbellar hypoplasia | Panel (448 genes): likely pathogenic variant: het HERC1 p.Arg2225; VOUS: het HERC1 p.Met1546Val | 25+5 | - | Live birth 40+4, LTFU |
| 16 | Complex CNS | No | M | 24+3 | CC hypoplasia, cerebellar hypoplasia, choroid plexus cyst | Trio ES: het (dn) TUBB mutation (C.670G>T.p.(Asp224Tyr)), OMIM #615771 | 26+3 | CDCBM6 | Live birth 38+5, LTFU |
| 17 | Complex CNS | No | M | 21+6 | Microcephaly, pACC, cerebellar and vermian hypoplasia, pachygyria-polymicrogyria complex | Panel (270 genes): compound het (mat) CENPJ mutation c.879dup p.Cys294Metfs*14, OMIM #251200; VUS: het (pat) c.3533G>C p.Arg1178Pro | 25+5 | MCPH | Live birth 39+1, LTFU |
| 18 | Complex CNS | No | M | 23+3 | Lissencephaly, pACC, mild VM | CMA: inconspicuous. Trio ES: hemi (mat) NSDHL mutation c.686+5G>A, OMIM #300831 | 27+0 | CK syndrome | TOP |
| 19 | Complex CNS | Yes | M | 20+1 | Severe hydrocephalus, pACC, isolated vermian hypoplasia, lissencephaly | CMA: inconspicuous. Panel (6700 genes): het (dn) TUBB3 mutation (NM_006086.4, c.1138C>T (p-Arg380Cys)), OMIM #614039 | - | CDCBM1 | Live birth 37+1, follow-up at four years: GDD, unable to speak and walk. Mental retardation with a delay of two years. MRI: stable, no shunting. |
| 20 | Complex CNS | No | M | 21+2 | Moderate VM, cerebellar hypoplasia, CC hypoplasia | Trio ES: het 6q26q27 deletion, 7 Mb, comprising 33 genes, including DLL1-gene, ORPHA: 75857 | Postnatal | Terminal 6q deletion syndrome | TOP |
| 21 | Complex CNS | No | F | 31+2 | Microcephaly, lissencephaly. FGR. | Trio ES: hom ASPM mutation c.9190C>T p.Arg3064* on chromosome 1, OMIM #608716 | - | MCPH5 syndrome | TOP |
| 22 | Complex CNS | No | M | 22+0 | Microcephaly, lissencephaly. | Trio ES: het (dn) GRIN1 mutation c.2003A>G, p.Tyr668Cys on chromosome 9. | - | - | TOP |
| Abbreviations: ACC, agenesis of corpus callosum; CC, corpus callosum; CNS, central nervous system; CDCBM1/6, complex cortical dysplasia with other brain malformations-1/-6; CMA, chromosomal microarray analysis; CK, creatine kinase; dn, de novo; consang., consanguinity; dup, duplication; Dx, diagnosis; ES; exome sequencing; F, female; FGR, fetal growth retardation; GA, gestational age; GDD, global developmental delay; hemi, hemizygous; het, heterozygous; hom, homozygous; LTFU, lost to follow up; M, male; mat, maternal; MCPH/5, autosomal recessive primary microcephaly/-5; NF1, neurofibromatosis type 1; pACC, partial ACC; panel, targeted gene panel analysis; TOP, termination of pregnancy; VM, ventriculomegaly; VUS, variant of unknown significance. | | | | | | | | | |

| **Table S2** - Distribution of structural CNS anomalies. | | | | | | |
| --- | --- | --- | --- | --- | --- | --- |
| CNS anomaly | Malformation-subtype | Total | Isolated CNS | Isolated CNS plus | Complex CNS | Multisystem  CNS |
| Ventriculomegaly (n= 481) | Mild | 133 | 20 | 14 | 21 | 78 |
|  | Moderate | 120 | 21 | 13 | 15 | 71 |
|  | Severe | 228 | 57 | 13 | 56 | 102 |
| Midline defects (n= 365) |  |  |  |  |  |  |
|  |  |  |  |  |  |  |
| Corpus callosum anomalies | Partial agenesis | 52 | 5 | 4 | 13 | 30 |
|  | Complete agenesis | 178 | 38 | 11 | 37 | 92 |
|  | Hypoplasia | 29 | 2 | 0 | 11 | 16 |
|  | Other pathologies | 5 | 0 | 0 | 2 | 3 |
| Abnormal cavum septi pellucidi |  | 17 | 0 | 0 | 7 | 10 |
| Holoprosencephaly | Lobar | 14 | 0 | 0 | 2 | 12 |
|  | Semilobar | 13 | 0 | 0 | 2 | 11 |
|  | Alobar | 49 | 2 | 0 | 3 | 44 |
|  | Interhemispheric variant | 8 | 0 | 0 | 2 | 6 |
| Posterior fossa anomalies  (n= 270) | Cerebellum anomaly | 85 | 3 | 1 | 20 | 61 |
|  | Dandy-Walker complex | 91 | 8 | 4 | 18 | 61 |
|  | Vermian anomaly | 21 | 0 | 0 | 5 | 16 |
|  | Rhombencephalosynapsis | 14 | 0 | 0 | 8 | 6 |
|  | Megacisterna magna | 34 | 4 | 0 | 5 | 25 |
|  | Persistent Blake's pouch cyst | 16 | 1 | 1 | 2 | 12 |
|  | Arachnoid cyst | 9 | 0 | 0 | 4 | 5 |
| Cortical malformations  (n= 190) |  |  |  |  |  |  |
| Proliferation disorders | Microcephaly | 74 | 0 | 0 | 20 | 54 |
|  | Hemimegalencephaly | 2 | 0 | 0 | 1 | 1 |
| Migration disorders | Lissencephaly | 88 | 0 | 1 | 35 | 52 |
|  | Heterotopia | 9 | 0 | 0 | 6 | 3 |
| Organisation disorders | Polymicrogyria | 7 | 0 | 0 | 5 | 2 |
|  | Schizencephaly | 10 | 1 | 0 | 6 | 3 |
| Others^a^ (n= 51) |  | 51 | 0 | 1 | 19 | 31 |
| Intracranial cysts^b^ (n= 36) |  | 36 | 1 | 0 | 20 | 15 |
| Brain stem anomalies (n= 9) | | 9 | 0 | 0 | 3 | 6 |
| The total number of anomalies exceeds the number of fetuses (n=780) as fetuses could present with more than one anomaly.  Abbreviations: CNS, central nervous system.  ^a^including encephaloceles, hydranencephaly and brain tumors.  ^b^excluding cysts of the posterior fossa. | | | | | | |
